# Lower extremity joint compensatory effects during the first recovery step following slipping and stumbling perturbations in young and older subjects

**DOI:** 10.1186/s12877-022-03354-3

**Published:** 2022-08-10

**Authors:** Xiping Ren, Christoph Lutter, Maeruan Kebbach, Sven Bruhn, Rainer Bader, Thomas Tischer

**Affiliations:** 1 College of Physical Education and Health Sciences, Zhejiang Normal University, 688 Yingbin Road, Jinhua, 321000 China; 2 Biomechanics and Implant Technology Research Laboratory, Department of Orthopaedics, Rostock University Medical Centre, Doberaner Strasse 142, 18057 Rostock, Germany; 3 Institute of Sport Science, University of Rostock, 18051 Rostock, Germany

**Keywords:** Aging, Treadmill perturbation, First recovery step, Spatiotemporal parameters, Joint dynamics

## Abstract

**Background:**

The lower extremity may play a crucial role in compensating for gait perturbations. The study aimed to explore the mechanism of perturbation compensation by investigating the gait characteristics and lower extremity joint moment effects in young (YS) and older subjects (OS) during the first recovery gait following slipping (slipping_Rec1) and stumbling (stumbling_Rec1).

**Method:**

An automatic perturbation-triggered program was developed using D-Flow software based on the Gait Real-time Analysis Interactive Lab to induce the two aforementioned perturbations. Marker trajectories and ground reaction forces were recorded from 15 healthy YS (age: 26.53 ± 3.04 years; body height: 1.73 ± 0.07 m; body mass: 66.81 ± 11.44 kg) and 15 healthy OS (age: 68.33 ± 3.29 years; body height: 1.76 ± 0.10 m; body mass: 81.13 ± 13.99 kg). The Human Body Model was used to compute the variables of interest. One-way analysis of variance and independent samples t-test statistical analyses were performed.

**Results:**

In slipping_Rec1 and stumbling_Rec1, the change in gait pattern was mainly reflected in a significant increase in step width, no alterations in step length and stance/swing ratio were revealed. Based on perturbed task specificity, lower extremity joint moments increased or decreased at specific phases of the gait cycle in both YS and OS in slipping_Rec1 and stumbling_Rec1 compared to normal gait. The two perturbed gaits reflected the respective compensatory requirements for the lower extremity joints, with both sagittal and frontal joint moments producing compensatory effects. The aging effect was not reflected in the gait pattern, but rather in the hip extension moment during the initial stance of slipping_Rec1.

**Conclusions:**

Slipping appears to be more demanding for gait recovery than stumbling. Gait perturbation compensatory mechanisms for OS should concentrate on ankle strategy in the frontal plane and counter-rotation strategy around the hip.

## Background

More than a quarter of the older population (over 65 years old) fall at least once a year [1], and the percentage is gradually rising, which seriously affects the quality of life of older subjects (OS) and imposes a huge socioeconomic burden on health care [2]. Unpredictable slipping and stumbling-induced fall accidents during walking are the most common mechanisms and are the leading cause of serious injuries in OS [3, 4]. OS have a reduced ability to cope with unpredictable gait perturbations relative to young subjects (YS) [5], possibly related to decreased muscle strength, flexibility, and stability [6]. Controlling dynamic gait stability after slipping and stumbling perturbations has therefore become a key area of concern in recent years.

The loss of equilibrium is generally followed by recovery stepping. Increased rates of falls are considerably associated with a lack of ability to regain one’s balance by taking restorative steps [7]. Humans can quickly regain stability and maintain balance from the same type of perturbation [8]. The “first-trial effect” has been used to describe the training effects of older adults experiencing the first slipping perturbation, suggesting that first-exposure trial generate rapid adaptive effects, and such effects could be maintained for up to a full year [9, 10]. The first response of a slipping perturbation has the largest effect on the gait variables compared with the subsequent perturbations of the same type [5, 11], and the first step following the perturbation is the most important protective strategy [12, 13]*.* A longer length of the first recovery step following a backward slipping [14] or a larger distance between the center of mass (CoM) and the recovery step after stumbling [15] plays a critical role in balance regaining. Therefore, quantifying the gait performance of recovery steps is intuitively critical following an unexpected slipping or stumbling perturbation. Successful stepping strategies could provide additional limb support to maintain the CoM within an effective base of support (BoS), thereby reducing the likelihood of falls [7]. This typically needs the successful application of dynamic stability control mechanisms, including modulation of stepping [16–18], activation of muscle moments around ankle [19] and changing the angular momentum around the hip [20, 21] and so on. These mechanisms are named as “stepping strategy”, “ankle strategy”, “counter-rotation strategy”, and “braking strategy” [22]. Some valuable variables such as the CoM [23, 24], the margin of stability (MoS) [23–25], the center of pressure (CoP) [23, 26] and the variability of joint angle [27] have been applied to gait perturbation studies extensively in different subjects. Spatiotemporal gait parameters typically represent changes in gait patterns, while kinetic variables are thought to reflect the biomechanical strategy of perturbation recovery, with joint moments serving as key indicators [28]. The rapid development of lower extremity joint moments that attenuate may predetermine the recovery strategies available to humans in the event of slipping or stumbling.

Slipping perturbation caused more joint movement than stumbling perturbation in YS [4]. Moreover, OS compensated for muscle weakness by using lower extremity joint moments and muscle activation as well as increased muscle co-contraction after stumbling [29]. To date, only a small portion of research has assessed the patterns of changes in lower extremity joint moments following perturbations through time-series features of the entire gait cycle. Only two studies investigated the joint moment effects induced by backward slipping perturbation on the treadmill [8, 14]; however, the role of lower extremity joint moments in response to perturbations was inconsistent in the results.

The aforementioned studies are still insufficient to fully verify the changes in gait pattern and kinetic responses of the lower extremity joints, and the experimental protocols are not comprehensive enough, as evidenced by the timing of perturbation occurrence, the varying settings of perturbation intensity, and the majority of applied populations focusing on YS, with a small number of studies on OS over 65 years old with a risk of falling. To the best of our knowledge, there is a lack of comparative studies on age groups subjected to backward acceleration and forward deceleration perturbations on a treadmill [24, 25], as well as the instant effects and mechanisms of lower extremity compensation for both types of perturbations, especially in the first recovery gait (Rec1) following the perturbations.

Therefore, the aim of this study was to investigate the gait compensation strategies used by YS and OS to maintain stability in the slipping_Rec1 and stumbling_Rec1. We hypothesized that (1) increasing step width or decreasing step length to alter gait patterns may be the favored gait stabilization mechanism; (2) the compensatory effect of lower limb joint moments may involve multiple planes; (3) aging may affect the compensatory effects.

## Method

### Participants

Fifteen YS (age: 26.53 ± 3.04 years, body height: 1.73 ± 0.07 cm, body mass: 66.81 ± 11.44 kg) and fifteen OS (age: 68.33 ± 3.29 years, body height: 1.76 ± 0.10 cm, body mass: 81.13 ± 13.99 kg) were recruited. The number of participants was referenced to a recent study that showed that a target power of 0.8 involving one-dimensional data effects in biomechanical experiments can be achieved with 5–40 sample sizes [30]. Regarding walking on a split-belt treadmill, none of the participants had experience. The dominant leg for kicking was the right leg in all subjects. Inclusion criteria were no neurological dysfunction, no musculoskeletal disorders, and no history of falling in the past six months. Participants were asked to wear their own sports shoes and tight elastic pants to ensure comfort during the measurement. All experimental protocols were approved by the Ethics Committee of the Medical Faculty of the University of Rostock, Germany (No. A2019-0231), which met the requirements of the Declaration of Helsinki. Before the investigation, all individuals provided written informed consent.

### Experimental protocol

**Fig. 1:**
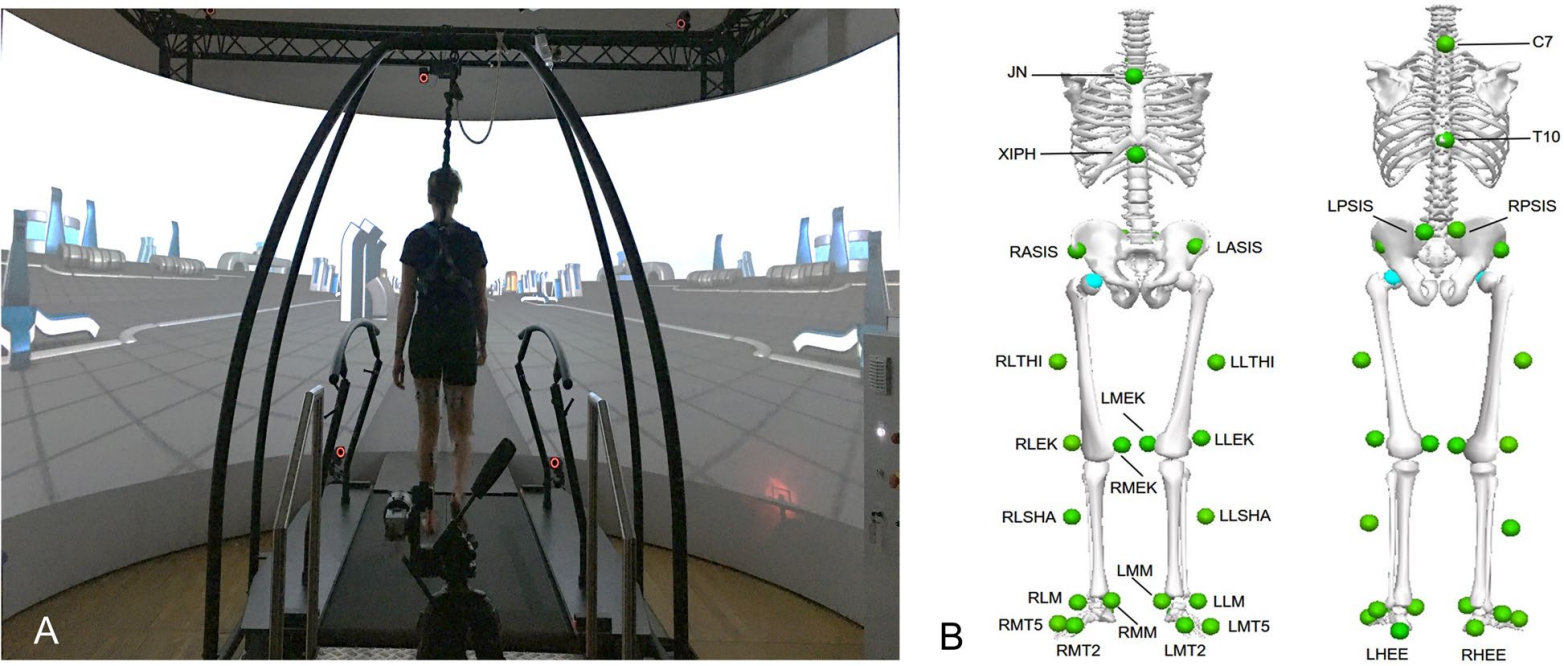
Exemplary trial with GRAIL and the scenario involved in this study (**A**); Front and rear view of the marker set used in the Human Body Model, with its 26 specific experimental skin markers, including the anatomical position of the lower extremities and trunk (**B**)

The experimental design was as in one of our latest study [31]. Briefly, the investigation was conducted at a Gait Real-time Analysis Interactive Lab (GRAIL, Motek, Houten, the Netherlands) (Fig. 1A). Marker trajectories were recorded by a 3D motion capture system with 10 high-resolution infrared cameras operating at 100 Hz (Vicon Motion System, Oxford, United Kingdom), and ground reaction forces (GRFs) were collected by a split-belt treadmill with two embedded force plates (Motek Force link, Houten, the Netherlands) operating at 1000 Hz. The scenarios presented by the 180° virtual reality screen made the participants feel like they were walking in real life, rather than on a treadmill. The whole system was controlled by D-flow (v3.34, Motek, Houten, the Netherlands) software with a custom script, which integrated all hardware in a synchronized fashion. The Human Body Model (HBM, v2.0, Motek, Houten, the Netherlands) was used to create a musculoskeletal model (Fig. 1B) [32]. The investigation consisted of three trials, including one initial familiarization, one normal walking, and one perturbation walking. Participants were instructed to try to recover after being perturbed and to continue walking without holding the handrail. The harness attached to an overhead frame was utilized to protect the participants’ safety and did not affect their walking.

**Fig. 2:**
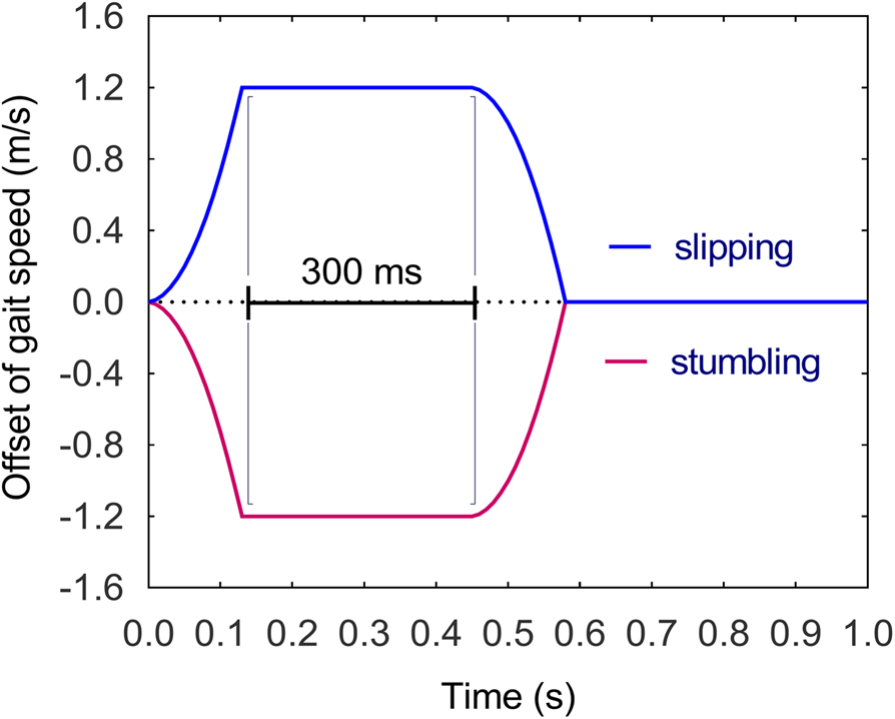
Slipping and stumbling perturbation profiles at a frame rate of 100 Hz were synchronized by D-Flow software based on kinematic acquisition frequency. All perturbations occurred at the moment of right heel strike with the belt. The slip perturbation was a belt acceleration of 3 m/s^2^, increasing 1.2 m/s over the subject’s normal gait velocity, holding for 300 ms, and then decelerating to normal velocity, while the fall perturbation was the opposite and decreased the velocity to a minimum value of 0. The schematic is the curve generated with the participant’s average velocity of 1.32 m/s. The x and y axes represent the time and speed offsets, respectively

The Timed Up and Go (TUG) test was performed to detect whether the participant had a potential propensity to fall [33], and a score of ≥ 13.5 s was utilized to identify individuals at higher risk of falling [34]. 26 retro-reflective markers with a diameter of 1.4 cm were then attached to the anatomical landmarks according to HBM2. Participants carried out a 6-min initial familiarization and warm-up on the treadmill [35] and their preferred walking speeds were recorded. During the experiment, participants walked continuously for two minutes without being perturbed in the first trial at their preferred speed. Then, pseudo-random perturbations were conducted in the following trial. Each participant experienced two different types of perturbations, namely a posterior acceleration (slipping) and an anterior deceleration (stumbling) of the treadmill belt at the moment of the right heel strike. The purpose of the slipping perturbation is to induce forward rotation and acceleration of the upper body relative to the lower body, resulting in a forward loss of dynamic stability, while the purpose of the stumbling perturbation is to induce backward rotation and deceleration of the upper body relative to the lower body, resulting in backward loss of dynamic stability. The intensity of perturbation was set to 3 m/s^2^ [25] and the offset was set to 1.2 m/s. The perturbation speed lasted for 300 ms when it accelerated or decelerated a specific value, and then reached to the participant’s preferred speed. The perturbation profile was shown in Fig. 2. Each perturbation was repeated six times. Washout between each perturbation was set to 15–20 s to ensure that participants had sufficient time to recover. The order of the perturbations was pseudorandomized but kept the same for all participants. Throughout the investigation, participants were not informed in advance of the occurrence and the point in time of the perturbation. The whole trial took around 15 min for each participant.

### Data processing

The HBM integrated into the Gait Offline Analysis Tool software (GOAT, v4.1, Motek, Houten, the Netherlands) was used to compute spatiotemporal parameters (i.e., step length, step width, percentage of stance phase (% stance), and gait speed) and joint moments involved in this study. The local maximum of the anterior–posterior position of the heel marker relative to the pelvis was used to determine the heel-strike event [36]. A 2nd order low-pass Butterworth filter with a 6 Hz cutoff frequency was set in the HBM since this was found to be the highest in kinematics related to gait [37]. To prevent artifacts in joint moments, GRFs were processed with the same filter cutoff as for marker trajectories [32]. To eliminate inter-subject variation to reduce the confounding effect, step length and joint moment were normalized by the participant’s leg length and body mass introduced by Hof [38], respectively.

Normal walking values were calculated and averaged over 20–25 consecutive strides for each participant [39]. It has been shown that participants’ gait rapidly improves their balance recovery when faced with the same type of repetitive perturbation [8]. To avoid anticipatory effects and to ensure the ecological validity of the perturbation, only the first gait following the first perturbation trial was included in this study for analysis.

### Statistical analyses

All parameters involved were tested for normal distribution using the Shapiro–Wilk test before determining an appropriate statistical method. The value of 0.05 was considered a sign of significance level.

Zero-dimensional variables were statistically analyzed by GraphPad Prism v8.0.2 (GraphPad software Inc., La Jolla, CA, USA). Unpaired samples t-tests [age, body height, body weight, Timed Up and Go (TUG) score, step length in normal gait and in stumbling_Rec1, step width in normal gait and in stumbling_Rec1, % stance in normal gait and in slipping_Rec1, gait speed in slipping_Rec1 and in stumbling_Rec1] and Mann–Whitney U-tests [body mass index (BMI), normal gait speed, step length in slipping_Rec1, step width in slipping_Rec1, % stance in tripping_Rec1, and gait speed in normal gait) were performed for comparisons between YS and OS along with Fisher’s exact test for gender.

One-way analysis of variance (ANOVA) with the Geisser-Greenhouse correction (step length and % stance in YS; step width in OS) and Friedman tests (step width in YS; step length and % stance in OS) were performed for the comparisons between normal gait, slipping_Rec1 and stumbling_Rec1. Tukey’s and Dunn’s approaches were used for post hoc multiple comparisons along with the above two methods, respectively.

One-dimensional continuous time series data of joint moment waveforms were statistically analyzed by MATLAB R2018b (The Mathworks, Natick, MA, USA) with Statistical Parameter Mapping 1D (SPM 1D, www. spm1d.org) approach, which was developed based on random field theory and has been described in detail elsewhere [40]. Independent samples t-tests were performed for the comparisons of joint moments between YS and OS. One-way ANOVA were performed for comparisons of joint moments in YS and OS for each of the three gait conditions, respectively. If the overall ANOVA reported significance, Bonferroni post hoc multiple comparisons were performed. A total of three pairwise analyses were conducted with an alpha adjustment of 0.05/3 = 0.017.

## Results

### Descriptive characteristics of subjects

Basic information on YS and OS is presented in Table 1. Mean body weight and BMI of OS was found to be significantly higher than YS (*p* = 0.005*; p* = 0.011); no significant difference in body height was found (*p* = 0.271). TUG value varied significantly (*p* = 0.001), but was not considered a fall risk in either group (TUG < 13.5). No significant differences were observed between the gait speed of the two groups (normal gait, *p* = 0.394; slipping_Rec1, *p* = 0.238; stumbling_Rec1, *p* = 0.213, respectively).

**Table 1:** Basic information (mean ± standard deviation and min–max ranges) from young subjects (YS) and older subjects (OS)

Variables	YS (*n* = 15)	OS (*n* = 15)	*p*-value
Age (years)	26.53 ± 3.04 (22, 31)	68.33 ± 3.29 (63, 77)	0.000***
Gender (male/female)	6/9	11/4	0.139
Body height (m)	1.73 ± 0.07 (1.64, 1.83)	1.75 ± 0.09 (1.58, 1.88)	0.271
Body mass (kg)	66.81 ± 11.44 (51.80, 88.60)	81.13 ± 13.99 (54.00, 108.30)	0.005**
BMI (kg/m2)	22.31 ± 2.80 (17.67, 27.04)	26.24 ± 4.76 (20.08, 37.41)	0.011*
TUG (s)	8.88 ± 0.72 (7.47, 10.08)	10.32 ± 1.23 (7.89, 12.70)	0.001***
Normal gait speed (m/s)	1.32 ± 0.13 (1.13, 1.62)	1.32 ± 0.14 (0.95, 1.48)	0.394
Slipping_Rec1 gait speed (m/s)	1.47 ± 0.14 (1.26, 1.78)	1.40 ± 0.16 (0.97, 1.64)	0.238
Stumbling_Rec1 gait speed (m/s)	1.23 ± 0.15 (0.98, 1.52)	1.30 ± 0.15 (0.97, 1.53)	0.213

Asterisks represents *p*-value classification with * *p* < 0.05, ** *p* < 0.01, *** *p* < 0.001, and **** *p* < 0.0001

### Gait variables of interest

**Fig. 3:**
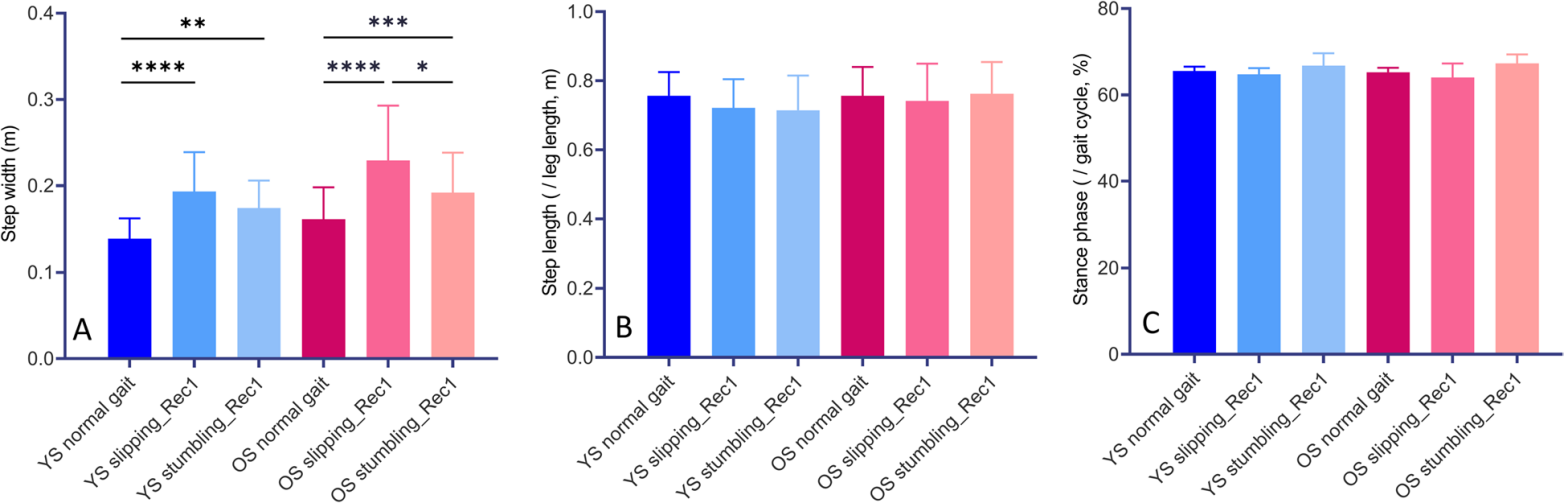
Step width (**A**), step length (**B**) and percentage of stance (**C**) for normal gait, slipping_Rec1 and stumbling_Rec1 in YS and OS. Asterisks represents *p*-value classification with * *p* < 0.05, ** *p* < 0.01, *** *p* < 0.001, and **** *p* < 0.0001

Friedman test and one-way ANOVA revealed significant differences in step width for three different gait conditions in YS (*p* < 0.0001) and OS (F (1.284, 17.98) = 19.42, *p* = 0.0002) (Fig. 3A). Specifically, the step width was significantly greater in slipping_Rec1 (*p* < 0.0001 for both YS and OS) and stumbling_Rec1 (*p* = 0.0042 for YS, and *p* = 0.0007 for OS) compared to normal gait; and it was greater in OS with slipping_Rec1 than with stumbling_Rec1 (*p* = 0.0449). We did not observe significant differences in step width between YS and OS for the same gait condition. Moreover, the step length (Fig. 3B), % stance (Fig. 3C) was not significantly different either in YS and OS for different gait conditions or between YS and OS for the same gait conditions.

### Ankle joint

**Fig. 4:**
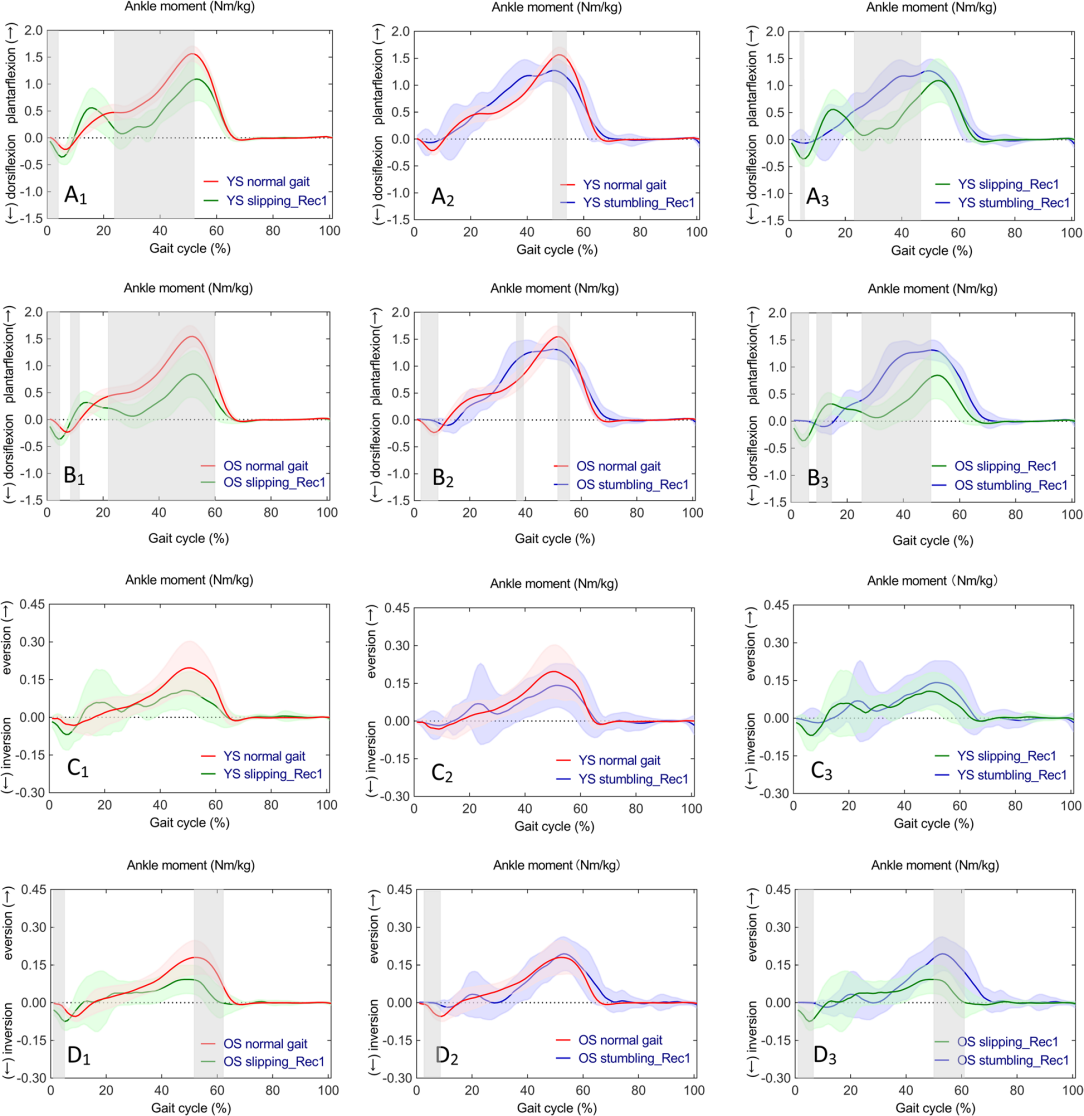
Time series curves of ankle moments in normal gait, slipping_Rec1, and stumbling_Rec1 in YS and OS, with different colored solid lines indicating the mean and corresponding-colored shaded areas indicating the standard deviation; and post hoc multiple comparisons test after one-way ANOVA, with gray bar areas indicating specific phase differences of gait cycle. A_1_-A_3_ and B_1_-B_3_ represent sagittal ankle moments in YS and OS; C_1_-C_3_ and D_1_-D_3_ represent frontal ankle moments in YS and OS

One-way ANOVA revealed statistically significant differences in continuous time series of sagittal ankle moments (F (2,42) = 8.136, *p* < 0.001, *p* < 0.001, and *p* = 0.048) and no significant differences in frontal ankle moments in YS in normal gait, slipping_Rec1, and stumbling_Rec1. Whilst, there were statistically significant differences in both sagittal and frontal ankle moments (F (2,42) = 8.103, *p* < 0.001 and *p* < 0.001; F (2,42) = 7.977, *p* < 0.001 and *p* < 0.001, respectively) in OS. Subsequent post hoc multiple comparisons are shown in Fig. 4 for sagittal ankle moments (Fig. 4A_1_, A_2_, A_3_ for YS; Fig. 4B_1_, B_2_, B_3_ for OS) and frontal ankle moments (Fig. 4C_1_, C_2_, C_3_ for YS; Fig. 4D_1_, D_2_, D_3_ for OS). A complete list of pairwise comparisons of ankle moments can be found in Table 2.

**Fig. 5:**
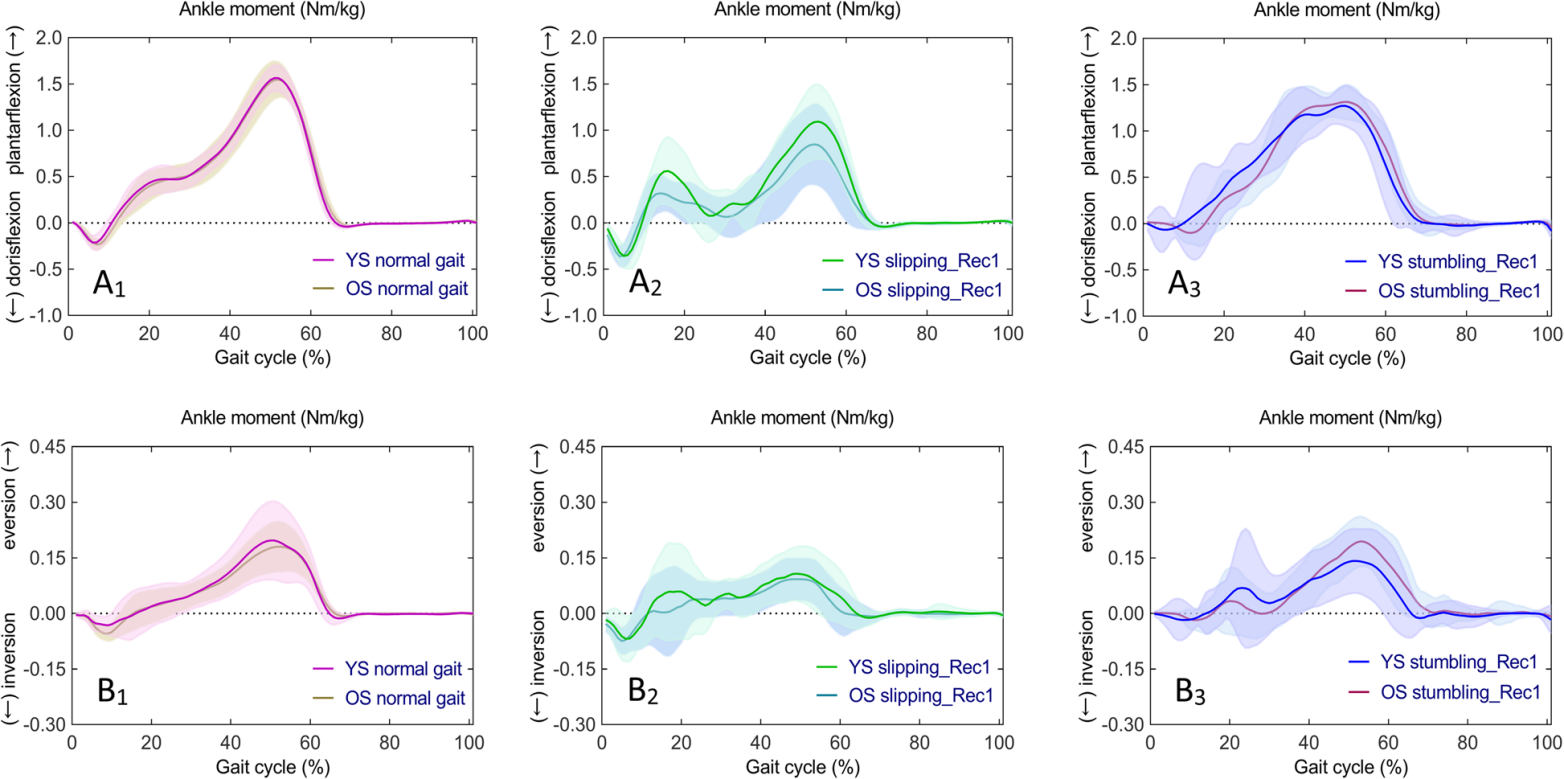
Time series curves of ankle moments in normal gait, slipping_Rec1, and stumbling_Rec1 between YS and OS, with different colored solid lines indicating mean values and corresponding-colored shaded areas indicating standard deviations; and results of independent sample t-test comparisons, with gray bar areas indicating specific phase differences of gait cycle. A_1_-A_3_ represent sagittal ankle moments between YS and OS; B_1_-B_3_ represent frontal ankle moments between YS and OS

**Fig. 6:**
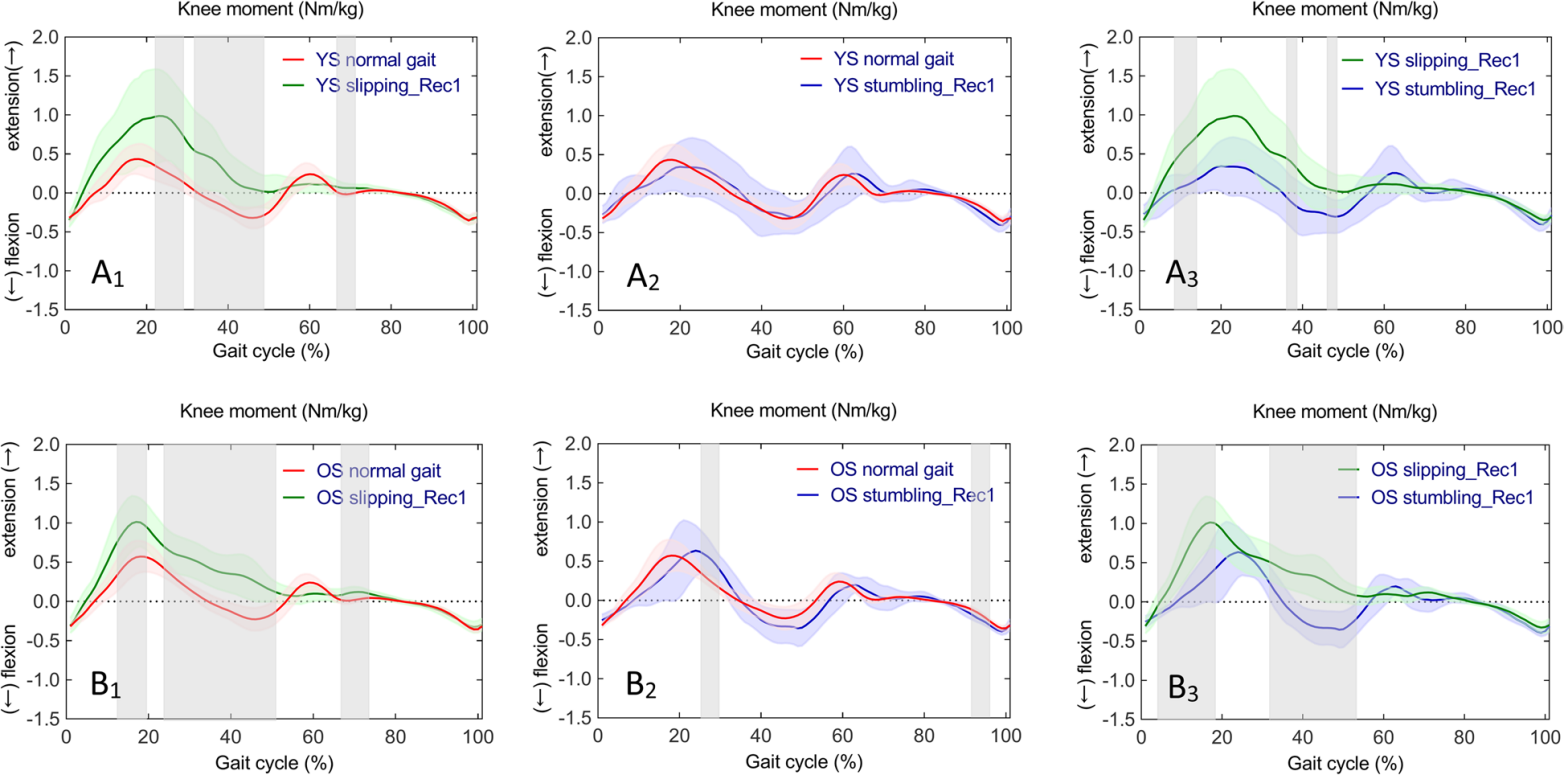
Time series curves of knee moments in normal gait, slipping_Rec1, and stumbling_Rec1 in YS and OS, with different colored solid lines indicating the mean and corresponding-colored shaded areas indicating the standard deviation; and post hoc multiple comparisons test after one-way ANOVA, with gray bar areas indicating specific phase differences of gait cycle. A_1_-A_3_ and B_1_-B3 represent sagittal knee moments in YS and OS

**Fig. 7:**
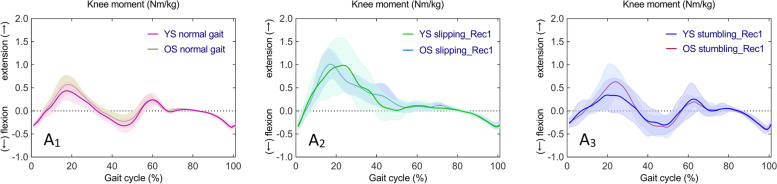
Time series curves of knee moments in normal gait, slipping_Rec1, and stumbling_Rec1 between YS and OS, with different colored solid lines indicating mean values and corresponding-colored shaded areas indicating standard deviations; and results of independent sample t-test comparisons, with gray bar areas indicating specific phase differences of gait cycle. A_1_-A_3_ represent sagittal knee moments between YS and OS

**Table 2:** Post hoc analysis (pairwise comparisons) results of ankle, knee, and hip moments for YS and OS. The ankle and hip moments are shown in both the sagittal and frontal planes, while the knee moment is only presented in the sagittal plane

Variable	Group	normal gait vs slipping_Rec1				normal gait vs stumbling_Rec1				slipping_Rec1 vs stumbling_Rec1			
		t*	n	cluster location	*p*-values	t*	n	cluster location	*p*-values	t*	n	cluster location	*p*-values
Sagittal ankle moment	YS	4.076	2	0–4.42%	*p* < 0.001	4.125	1	49.1–54.29%	*p* < 0.001	4.109	2	3.91–5.56%	*p* = 0.009
				23.67–51.83%	*p* < 0.001							23.48–46.04%	*p* < 0.001
	OS	4.082	3	0–4.34%	*p* < 0.001	4.140	3	0.86–8.09%	*p* < 0.001	4.105	3	0–6.07%	*p* < 0.001
				8.73–11.07%	*p* = 0.006			36.9–39.01%	*p* = 0.005			9.58–14.4%	*p* < 0.001
				22.19–59.44%	*p* < 0.001			52.16–55.52%	*p* = 0.001			25.38–49.48%	*p* < 0.001
Frontal ankle moment	YS	3.993	-	-	-	4.042	-	-	-	4.014	-	-	-
	OS	4.024	2	0–4.48%	*p* = 0.001	4.103	2	0–1.53%	*p* = 0.01	4.070	2	0–6.51%	*p* < 0.001
				51.79–61.78%	*p* < 0.001			3.01–8.81%	*p* < 0.001			50.01–60.53%	*p* < 0.001
Sagittal knee moment	YS	3.980	3	21.59–28.68%	*p* < 0.001	4.042	_	_	_	4.027	3	8.836–13.65%	*p* = 0.001
				31.69–48.98%	*p* < 0.001							36.46–38.02%	*p* = 0.012
				66.79–70.58%	*p* = 0.004							45.55–48.44%	*p* = 0.005
	OS	3.987	3	12.43–19.48%	*p* < 0.001	4.080	2	25.32–29.41%	*p* = 0.001	4.049	2	4.14–17.51%	*p* < 0.001
				23.61–50.55%	*p* < 0.001								
				66.94–73.26%	*p* < 0.001			91.66–95.63%	*p* = 0.001			32–53.1%	*p* < 0.001
Sagittal hip moment	YS	4.088	1	4.21–14.99%	*p* < 0.001	4.076	2	9.58–12.59%	*p* = 0.003	4.07	1	1.79–17.54%	*p* < 0.001
								46.31–51.91%	*p* < 0.001				
	OS	3.982	1	0–20.33%	*p* < 0.001	4.085	5	5.37–15.29%	*p* < 0.001	4.052	2	1.36–17.72%	*p* < 0.001
								37.8–38.14%	*p* = 0.017				
								42.79–44.63%	*p* = 0.009				
								46.18–52.43%	*p* < 0.001			33.47–51.49%	*p* < 0.001
								61.56–63.21%	*p* = 0.001				
Frontal hip moment	YS	3.984	_	_	_	4.020	2	11.55–21.57%	*p* < 0.001	4.014	1	9.49–24.68%	*p* < 0.001
								46.78–55.03%	*p* < 0.001				
	OS	3.944	1	6.19–25.03%	*p* < 0.001	4.025	2	0–1.58%	*p* = 0.012	4.003	2	0–1.58%	*p* = 0.012
								10.95–18.25%	*p* < 0.001			7.00–23.68%	*p* < 0.001

Presented are the t-threshold (t*), the number of clusters (n) and the location of cluster occurrences throughout the gait cycle, and the *p*-value for each cluster in the post hoc analysis. Non-significant results are indicated by small horizontal lines (-)

Specifically, compared to normal gait, ankle dorsiflexion moments increased during early stance, and ankle plantarflexion moments decreased from mid-stance to late stance in slipping_Rec1 in both YS and OS; ankle plantarflexion moments decreased during latestance in stumbling_Rec1 in both YS and OS as well as ankle dorsiflexion moments decreased during early stance in OS. Comparing the two perturbed gaits, ankle dorsiflexion moments were larger during early stance in slipping_Rec1 in both YS and OS as well as lower plantarflexion moments from mid-stance to late stance. Compared to normal gait, inversion moments increased during early stance as well as decreased eversion moments during late stance in slipping_Rec1 and inversion moments during early stance in stumbling_Rec1 in OS. Comparing the two perturbed gaits, there was a larger inversion moment during early stance and a smaller eversion moment during late stance in slipping_Rec1 in OS.

Both the sagittal and frontal ankle moment time series curves were similar for YS and OS in normal gait, slipping_Rec1, and stumbling_Rec1, with no statistically significant difference between the two groups according to the independent samples t-tests (Fig. 5A_1_, A_2_, A_3_, and Fig. 5B_1_, B_2_, B_3_, respectively).

### Knee joint

One-way ANOVA revealed statistically significant differences in continuous time series of knee moments (F (2,42) = 7.818, *p* < 0.001) in YS in normal gait, slipping_Rec1, and stumbling_Rec1. Whilst, there were statistically significant differences in knee moments (F (2,42) = 7.900, *p* < 0.001, *p* < 0.001, and *p* = 0.045) in OS. Subsequent post hoc multiple comparisons are shown in Fig. 6A_1_, A_2_, A_3_ for YS and Fig. 6B_1_, B_2_, B_3_ for OS. A complete list of pairwise comparisons of knee moments can be found in Table 2.

Specifically, compared to normal gait, knee extension moments increased during mid-stance, terminal-stance, and initial swing in slipping_Rec1 in both YS and OS; knee extension moments increased during mid-stance as well as knee flexion moments increased in stumbling_Rec1 in OS. Comparing the two perturbed gaits, there was a larger knee extension moment from early stance to mid-stance and a lower knee flexion moment from terminal stance to late stance in both YS and OS in slipping_Rec1.

The knee moment time series curves were similar between YS and OS in normal gait, slipping_Rec1, and stumbling_Rec1 (Fig. 7A_1_, A_2_, A_3_). Independent samples t-tests revealed no statistically significant differences in knee moments between YS and OS in slipping_Rec1 and stumbling_Rec1.

### Hip joint

**Fig. 8:**
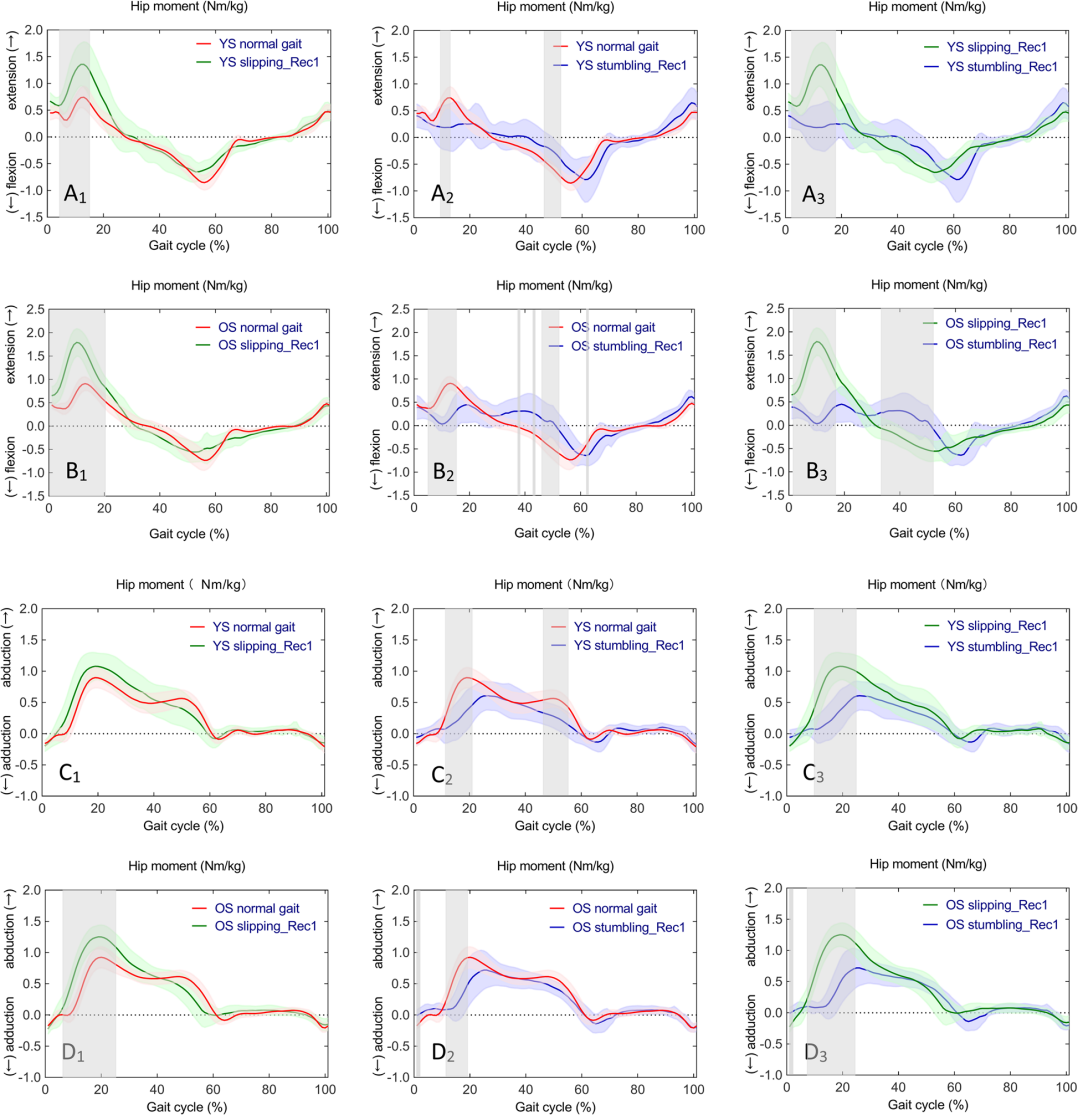
Time series curves of hip moments in normal gait, slipping_Rec1, and stumbling_Rec1 in YS and OS, with different colored solid lines indicating the mean and corresponding-colored shaded areas indicating the standard deviation; and post hoc multiple comparisons test after one-way ANOVA, with gray bar areas indicating specific phase differences of gait cycle. A_1_-A_3_ and B_1_-B_3_ represent sagittal hip moments in YS and OS; C_1_-C_3_ and D_1_-D_3_ represent frontal hip moments in YS and OS

One-way ANOVA revealed statistically significant differences in continuous time series of sagittal hip moments in YS (F (2,42) = 8.011, *p* < 0.001, *p* < 0.001, and *p* < 0.001) in normal gait, slipping_Rec1, and stumbling_Rec1. Whilst, there were statistically significant differences in sagittal hip moments in OS (F (2,42) = 7.915, *p* < 0.001, *p* < 0.001, *p* = 0.011, *p* = 0.047, and *p* = 0.050). Significant differences in frontal hip moments in normal gait, slipping_Rec1, and stumbling_Rec1 in YS (F (2,42) = 7.777, *p* = 0.048, *p* = 0.004, and *p* = 0.039) and in OS (F (2,42) = 7.723, *p* = 0.035, *p* = 0.028, and *p* = 0.048) were observed. Subsequent post hoc multiple comparisons are shown in Fig. 8 for sagittal hip moments (Fig. 8A_1_, A_2_, A_3_ for YS; Fig. 8B_1_, B_2_, B_3_ for OS) and frontal hip moments (Fig. 8C_1_, C_2_, C_3_ for YS; Fig. 8D_1_, D_2_, D_3_ for OS). A complete list of pairwise comparisons of hip moments can be found in Table 2.

**Fig. 9:**
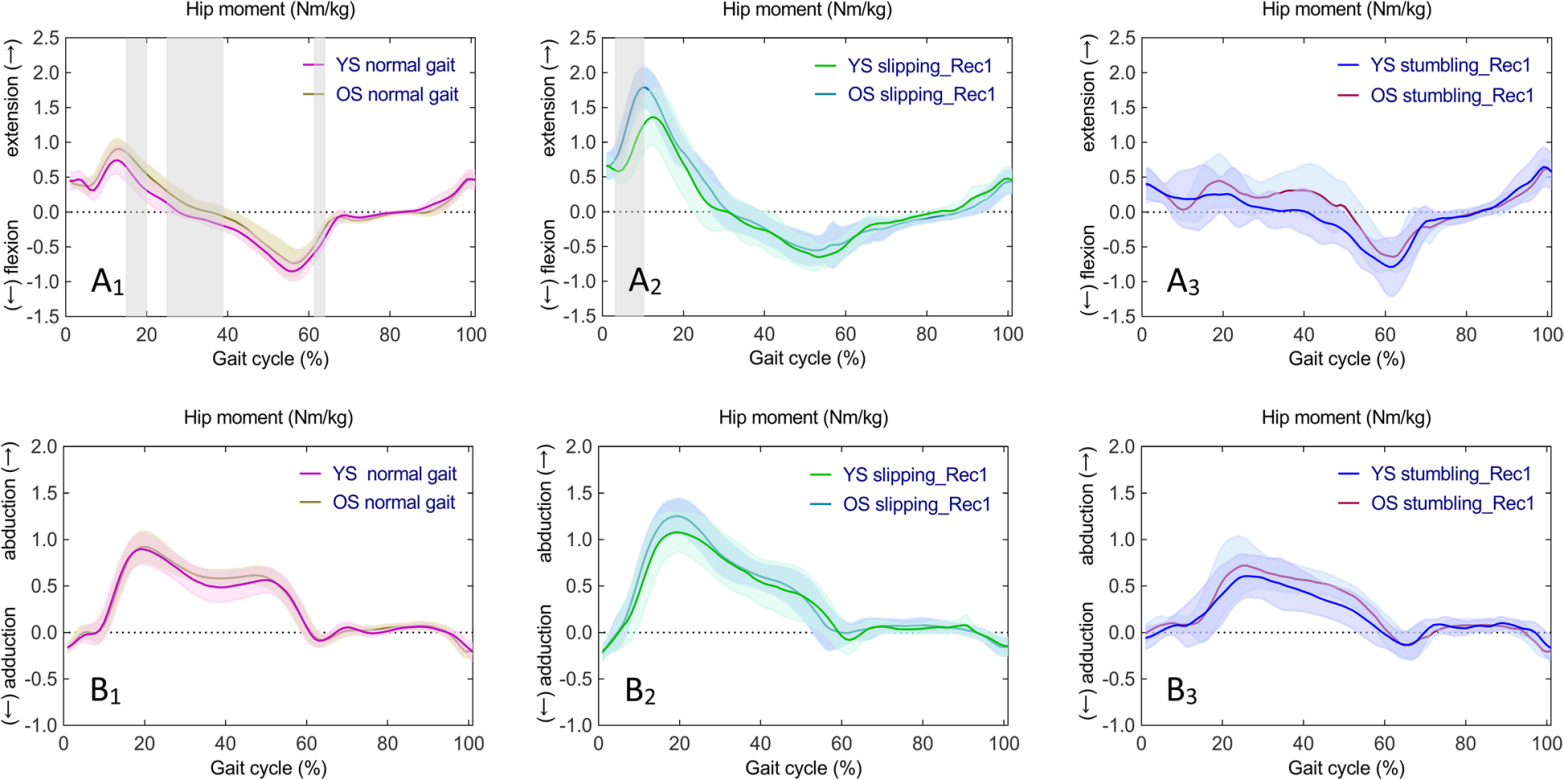
Time series curves of hip moments in normal gait, slipping_Rec1, and stumbling_Rec1 between YS and OS, with different colored solid lines indicating mean values and corresponding-colored shaded areas indicating standard deviations; and results of independent sample t-test comparisons, with gray bar areas indicating specific phase differences of gait cycle. A_1_-A_3_ represent sagittal hip moments between YS and OS; B_1_-B_3_ represent frontal hip moments between YS and OS

Specifically, compared to normal gait, hip extension moments increased from early stance to mid-stance in slipping_Rec1 in both YS and OS; hip extension moments decreased during early stance and hip flexion moments decreased during late stance in stumbling_Rec1 in both YS and OS. Comparing the two perturbed gaits, there was a larger hip extension moment from early stance to mid-stance in both YS and OS and a lower hip flexion moment during terminal stance in OS in slipping_Rec1. Compared to normal gait, abduction moments increased during mid-stance in slipping_Rec1 in OS but no differences in YS; abduction moments decreased during mid-stance in both YS and OS as well as during late stance in YS. Comparing the two perturbed gaits, there was a larger abduction moment during mid-stance in both YS and OS in slipping_Rec1.

The sagittal and frontal hip moment time series curves were similar between YS and OS in normal gait, slipping_Rec1, and stumbling_Rec1 (Fig. 9A_1_, A_2_, A_3_, and Fig. 9B_1_, B_2_, B_3_, respectively). Independent samples t-tests revealed statistically significant differences in sagittal hip moments between YS and OS in normal gait (*p* = 0.002 at 14.63–19.89%, *p* < 0.001 at 25.51–38.02%, and *p* = 0.003 at 61.83–63.90%, respectively, Fig. 9A_1_) and slipping_Rec1 (*p* < 0.001 at 3.32–10.05%, Fig. 9A_2_), respectively.

## Discussion

The main findings were that (1) the step width for YS and OS increased significantly in both slipping_Rec1 and stumbling_Rec1. In slipping_Rec1, OS had a significantly wider step than that of stumbling_Rec1. Our hypothesis 1 was partially confirmed that the change in step width is an essential strategy to maintain gait stability; (2) both sagittal and frontal joint moments produce compensatory effects. The frontal ankle joint strategy was well represented in OS, and (3) in response to the same perturbation, OS required a greater hip extension moment than YS to compensate for slipping perturbation during earlystance.

### Alteration of gait pattern

When a perturbation occurs, the cerebral cortex instinctively responds by adopting more cautious gait to position the body for stability [41]. Generally such gait alterations are referred to as gait placement strategies [17, 18].These deliberate modifications protect the dynamic stability and can lessen the requirement for time-critical, reactive control [9, 42]. As such, it is frequently employed by older adults [43]. Gait pattern alterations are typically characterized and quantified in terms of spatiotemporal parameters [44]. To proactively reduce anticipated disturbances, wider and shorter steps are regularly applied, which likewise works for unanticipated ones [42].Wider steps taken voluntarily help improve immediate lateral stability [45], and shorter step length can lead to an increase in the medio-lateral and backward MoS against anterior–posterior slip [46, 47]. These observable stepping strategies are an important contribution to fall prevention [46]. The essence is to shift the CoM closer to the BoS [47, 48].

In the present study, compared to normal gait, YS and OS had a significant increase in step width for both slipping_Rec1 and stumbling_Rec1, but no difference was found between YS and OS. The increase in step width is consistent with most previous studies [24, 45, 49]. The specificity of the perturbation task was demonstrated in OS, as reflected by the wider step width of slipping_Rec1.

Surprisingly, we did not observe any significant difference in step length, although there was a slight reduction for both YS and OS. This finding is supported by a recent study, which revealed that a key element in the stability of perturbation recovery is to keep the step length of Rec1 close to normal [14], as increasing step length could compensate for greater CoM shifts [14, 45]. However, this seems to be inconsistent with some previous studies that have concluded that shorter step length improves gait stability against slipping perturbations in the anterior–posterior direction [46, 47]. This could be related to the procedure of the perturbation setup. Expected and unanticipated perturbations can produce inconsistent effects. Studies showed that human, however, subconsciously reduce step length when subjected to warning perturbations [42, 47], which is a sign of “caution” [45]. Moreover, a recent study concluded that the changing gait patterns caused adjustments in Stance/Swing ratio, thus shortening the stance time, which was also considered to be one of the main strategies for recovery after backward slipping perturbation [50]. Our results are inconsistent with the above findings, where the percentage of stance did not change significantly in both slipping_Rec1 and stumbling_Rec1. This should be related to the fact that Rec1 following perturbations consistently maintained relatively high stability in the anterior–posterior direction based on step length. Since the current study implemented perturbations in the anterior–posterior direction, no medial–lateral perturbations occurred and it was the first perturbation that was unexpected and reflected the true human response. Sudden and unanticipated perturbations did not produce a learning effect, so there could be maintenance of dynamic stability in multiple planes [18, 51]. Humans subjected to mechanical perturbations of gait do not dramatically reposition the foot in the sagittal plane, but do relocate the CoP to counteract the effects of perturbations after foot placement. Instead, the CoP primarily reflects the utilization of sagittal and frontal ankle joint moments [16, 20, 52].

### Effects on lower extremity joint moment

The role of joint moments to balance recovery is task-dependent, as mechanical requirements vary considerably [8, 14, 51]. The slipping perturbation in our investigation triggered the right belt to accelerate backward, resulting in a forward loss of dynamic stability. Debelle et al. conceptualized the mechanics of the recovery steps after a forward-falling slipping as being mainly made up of two phases: avoidance of falls and recovery of balance, with fall avoidance including the propulsion phase of slipping and loading phase of Rec1, and balance recovery including the propulsion phase of Rec1 and return to normal from the loading phase of Rec2 [8]. Nevertheless, variables that differ from normal during Rec2 were not linked to dynamic stability [14]. Compared to normal gait in YS, higher hip extensor moments were needed in slipping_Rec1 to avoid the fall and larger knee extensor moments were needed to raise and advance the CoM during Rec1, but there were no dominant effects on ankle joint moments [8]. YS and OS also developed a lower ankle plantarflexion moment in Rec1 than in normal gait [14]. This was consistent with the current study. Moreover, we observed that in OS, ankle inversion moments in slipping_Rec1 increased significantly in early stance and decreased significantly in late stance, as well as hip abduction moments significantly increased in mid-stance, suggesting that OS attempt to shift GRFs to reposition the CoP. This is a typical strategy for controlling medio-lateral stability, termed the ankle strategy [17, 52, 53]. However, we did not observe such results in YS. Liu [54]reported that the ankle and knee joints were essential in regulating gait perturbation in the sagittal plane, whereas the hip joint was primarily responsible for sustaining upper body balance in the frontal plane. Our findings further extend the studies by emphasizing the importance of ankle and hip joint adjustment for gait perturbations in the sagittal and frontal planes, especially in OS. However, it is essential to note that our study differs from the two aforementioned studies in the timing of perturbation onset [8, 14]. Specifically, the slipping perturbation occurred at heel strike in the current study, while perturbations were induced at the 20% stance phase in the other two studies. The different perturbation triggering time effects deserve further attention in subsequent studies. In addition, the stumbling perturbation in our investigation triggered the belt to decelerate forward, resulting in a backward loss of dynamic stability. This study indicated that knee joint moment in YS did not differ between the stumbling_Rec1 and normal gait, which was consistent with a previous study [4]. However, significant differences were observed in OS. Significant differences existed for the sagittal ankle and hip joint moments between stumbling_Rec1 and normal gait in both YS and OS, whereas no differences were observed between the two groups. We further observed that in OS, the ankle inversion moment was significantly reduced in early stance and the hip abduction moment was significantly reduced in mid-stance, while the abduction moment in YS was reduced in both mid-stance and late stance in stumbling_Rec1.

Our study indicated that ankle, knee, and hip joint moments of Rec1 were significantly different between slipping and stumbling perturbations in YS and OS. Recovery from slipping is likely to be more difficult than that from stumbling [8]. This is consistent with these three studies, of which one concerns forward falling slipping and the other two backward falling slipping [4, 55]. However, the study of Timsina et al.stated that increased foot clearance variability in OS led to an increased probability of stumbling, and stumbling perturbations cause more fall-related injuries than slipping perturbations, but without elucidating the specific biomechanical mechanisms [56]. Furthermore, Roeles et al. showed that stumbling perturbations caused the largest MoS difference compared to other types of perturbation in the anterior–posterior direction and required more recovery steps [24]. Therefore, stumbling perturbation was thought to be the most challenging. Despite the same perturbation intensity being used to trigger slipping and stumbling in our study, the results showed that the joint moments in Rec1 following the perturbation were greater for slipping than for stumbling. This necessarily requires more muscle involvement. Further investigation is needed to determine which type of perturbation is more difficult and suitable for detecting poor gait stability. Such work needs to involve perturbation recovery mechanisms, including a combination of dynamics, electromyography, and simulation.

Hip moments differed between YS and OS in normal gait and slipping_Rec1, with OS having relatively higher extensor moments. This indicates that OS require greater compensation in response to the same intensity of slipping. This was inconsistent with a recent study [14], which demonstrated that OS showed the same improvements as YS in response to slipping. To the best of our knowledge, this could be the most recent finding for the treadmill belt posterior acceleration perturbation (slipping). One potential explanation is that although BMI was similar between the two studies, the difference might be related to the age of the recruited OS, 68.33 ± 3.29 years (*n* = 15) in this study compared to 62.40 ± 6.60 years (*n* = 17) in the aforementioned study. The capacity to produce large anterior balance restoration steps decreases with aging [21], especially for older adults over 65 years of age. This requires the development of larger internal moments in the hip and knee joints [8]. In a study of slipping recovery in YS, it was found that hip joint always played the most important role, regardless of the slipping recovery strategies [57]. Typically, muscles generate force moments across joints during walking [58]. Muscle weakness of the lower extremities is an important risk factor for falls in OS [59] and decreasing joint power with aging seems to increase the risk of falls [60]. Moreover, muscles surrounding the hip were discovered to be the most significant in minimizing stumbling perturbation responses [61]. Therefore, it is necessary to focus on the strength of the flexion and extension muscles surrounding hip in OS. Future studies need to incorporate EMG and further use musculoskeletal multi-body simulations to investigate the differences in muscle strength between OS and YS during backward slipping perturbations, which might be an interesting aspect. Another potential explanation is the counter-rotation mechanism of gait stabilization conditioning, defined by Horak and Nashner [19] as a hip strategy based on kinematic characteristics, i.e., a reverse rotation around the upper and lower body of the hip joint. In the present study, slipping perturbation occurred mainly in the sagittal plane, i.e., in the anterior–posterior direction. It is reflected by an anterior acceleration of the torso in the flexion direction, which leads to a posterior acceleration of the CoM. During the loading response of the first stepping following a posterior slipping perturbation, the counter-rotation mechanism counteracts the acceleration of the CoM to prevent gait disturbances [17] with the aim of altering the direction of the GRF by changing the angular momentum of the limb segments around the CoM [20]. The angular momentum range becomes significantly larger when the gait stability is disrupted by disturbances [62], and this increase may differ in YS and OS, leading to differences in hip extension moments.

The experimental data suggested that the counter-rotation mechanism prevents disturbance of the gait pattern, but only during the early phase of the slipping_Rec1 [17], which is consistent with our findings. Notably, the magnitude of joint moments is strongly dependent on gait speed, as indicated by the fact that increasing gait speed raises peak values of moments and alters the pattern [63–65]. In the present study, there were no statistically significant differences in gait speed between YS and OS, so it should be valuable to use the counter-rotation mechanism to explain the differences in hip moment between the two groups, reflecting the need for the hip extensors in OS to generate a greater extension moment to achieve counter-rotation control of speed. Undoubtedly, how gait speed affects the adaptation of joint moments to reactive recovery after perturbation in different age and patient populations remains a question for future research [66]. An approach that adjusts gait speed to an equivalent MoS to reduce inter-individual differences in gait stability may address this issue [67]. This deserves further confirmation in our subsequent studies.

There are some limitations of this study that need to be mentioned. First, gender may have an effect on lower extremity kinematics and kinetics in the sagittal plane due to gender features of the gait-related anatomy. It was found that women had larger hip flexion and smaller knee extension before heel-strike as well as the higher knee flexion moment during pre-swing [68]. At standardized self-selected speeds, hip extension moments were greater in women than in men [69]. Thus, the averaging of kinetic time series data may have weakened the female characteristics due to the higher proportion of males in OS. Second, the intensity of acceleration and deceleration perturbation was set to 3 m/s^2^. To date, there is no standardized perturbation intensity in previous studies [66]. To improve the balance response when walking, even minor perturbation magnitudes can elicit stepping reactions in OS and these responses are highly comparable to perturbations elicited by larger magnitudes [70], especially during the first step following perturbations [17]. Instead, when the perturbations occurred, participants were able to compensate with recovery steps, so that we could derive the compensatory mechanism for Rec1. The intensities used in this study were designed to avoid inducing falls, which we believe is desirable in clinical practice, although greater intensities may be needed to detect small group differences. Third, the HBM model is developed primarily for clinical applications and only the sagittal plane is considered for the knee joint; the other two dimensions of the frontal and horizontal planes are not represented. With the rapid development of computer technology, the current model might be further expanded to achieve multi-dimensional calculations of the knee joints in the future, which would be valuable for studying the mechanisms of gait stability control in elderly patients, such as those with knee osteoarthritis.

## Conclusions

Humans may compensate for slipping and stumbling perturbations simultaneously through multiple mechanisms to ensure stable recovery, such as stepping strategy, ankle strategy, and counter-rotation mechanisms. Gait recovery due to slipping appears to be more demanding than that of stumbling. In slipping_Rec1 and stumbling_Rec1, the step widths of YS and OS increased significantly and joint moments produce changes at different phases of gait cycle. The aging effect was mainly reflected in the fact that OS had a greater hip extension moment than YS, which is demonstrated during the initial phase of slipping_Rec1. The role of hip flexion and extension function in preventing perturbations in OS needs further attention. Our findings could lead to a better understanding of the underlying biomechanical mechanisms of belt acceleration and deceleration-induced perturbations on the treadmill.

## Data Availability

The data is not available publicly due to privacy. However, the datasets generated during the current study are available from the corresponding author upon reasonable request.

## Abbreviations

CoP: Center of pressure
OS: Older subjects
YS: Young subjects
Rec1: The first recovery step
Slipping_Rec1: The first recovery step following slipping
Stumbling_Rec1: The first recovery step following stumbling
CoM: Center of mass
BoS: Base of support
GRFs: Ground Reaction Forces
TUG: Timed Up and Go
GRAIL: Gait Real-time Analysis Interactive Lab
HBM: Human Body Model
GOAT: Gait Offline Analysis Tool
SPM: Statistical Parametric Mapping
ANOVA: Analysis of variance
